# Research on a nondestructive model for the detection of the nitrogen content of tomato

**DOI:** 10.3389/fpls.2022.1093671

**Published:** 2023-01-11

**Authors:** Xiaodong Zhang, Chaohui Duan, Yafei Wang, Hongyan Gao, Lian Hu, Xinzhong Wang

**Affiliations:** ^1^College of Agricultural Engineering, Jiangsu University, Zhenjiang, China; ^2^Key Laboratory of Modern Agricultural Equipment and Technology, Ministry of Education, Jiangsu University, Zhenjiang, Jiangsu, China; ^3^Key Laboratory of Key Technology on Agricultural Machine and Equipment, Ministry of Education, South China Agricultural University, Guangzhou, China

**Keywords:** terahertz spectroscopy, tomato, N, characteristic band, nondestructive detection

## Abstract

The timely detection of information on crop nutrition is of great significance for improving the production efficiency of facility crops. In this study, the terahertz (THz) spectral information of tomato plant leaves with different nitrogen levels was obtained. The noise reduction of the THz spectral data was then carried out by using the Savitzky-Golay (S-G) smoothing algorithm. The sample sets were then analyzed by using Kennard-Stone (KS) and random sampling (RS) methods, respectively. The KS algorithm was optimized to divide the sample sets. The stability competitive adaptive reweighted sampling (SCARS), uninformative variable elimination (UVE), and interval partial least-squares (iPLS) algorithms were then used to screen the pre-processed THz spectral data. Based on the selected characteristic frequency bands, a model for the detection of the nitrogen content of tomato based on the THz spectrum was established by the radial basis function neural network (RBFNN) and backpropagation neural network (BPNN) algorithms, respectively. The results show that the root-mean-square error of correction (RMSEC) and root-mean-square error of prediction (RMSEP) of the BPNN model were respectively 0.1722% and 0.1843%, and the determination coefficients of the correction set (*R_c_*^2^) and prediction set (*R_p_*^2^) were respectively 0.8447 and 0.8375. The RMSEC and RMSEP values of the RBFNN model were respectively 0.1322% and 0.1855%, and the *R_c_*^2^ and *R_p_*^2^ values were respectively 0.8714 and 0.8463. Thus, the accuracy of the model established by the RBFNN algorithm was slightly higher. Therefore, the nitrogen content of tomato leaves can be detected by THz spectroscopy. The results of this study can provide a theoretical basis for the research and development of equipment for the detection of the nitrogen content of tomato leaves.

## 1 Introduction

China is the world’s largest tomato-planting country, accounting for about 1/3 of the global tomato-planting area (Wang et al., 2021). At present, the planted area of facility tomato in China is in a leading position globally, but there is a large gap between the per-mu yield of facility tomato in China and that in developed countries. The main reason for this is that China has some long-term problems growing crops. The misuse of fertilizer during production leads to soil pollution, and a lack of a timely understanding of the nutritional status of crops leads to shortages of nutrients and the water supply during the fertilizer season (Li et al., 2019; Hu and Cai, 2021). Therefore, to improve the production efficiency of facility crops and avoid environmental problems caused by the unreasonable application of chemical fertilizers, it is necessary to carry out scientific and theoretical research on the detection of information on crop nutrition. This is expected to greatly improve the production efficiency of facility crops and reduce pollution. Thus, technology for the non-destructive testing of crop nutrition is of great significance.

In the traditional facility nutrition management process, the judgment of the nutritional status of plants is mainly realized by expert experience and chemical determination, which are characterized by some problems. Expert experience is easily affected by subjective factors, and accurate judgment cannot be achieved (Fitzgerald et al., 2006; Huang et al., 2009; Zhang et al., 2018). Although chemical determination has high detection accuracy, it is difficult to realize the dynamic feedback control of crop nutrition information due to poor timeliness, and the sampling process causes certain damage to crops (Gao et al., 2012; Tian et al., 2016).

In recent years, non-destructive testing technology has been used to diagnose the nutritional elements of crops. This technology can quickly judge the nutritional status of crops without causing damage to them, and has gradually become a popular method for nutritional testing (Song et al., 2016; Yang et al., 2018). Some research has been conducted on the nutritional element diagnosis of crops both domestically and internationally, and some achievements have been made; however, there remain some shortcomings. For example, the method of crop information processing is relatively simple, and the model accuracy is not high (Yada et al., 2008; Hang et al., 2015). Moreover, related research is mainly focused on the analysis of the reflection intensity, texture, and other characteristics of the crop leaves, and biological macromolecules inside crops, such as nucleic acids and phospholipids, cannot be detected in detail (Breitenstein et al., 2012; Gente et al., 2013; Gente et al., 2015).

Terahertz (THz) detection technology an advanced technology known as “one of the ten technologies that will affect the future of mankind in the 21^st^ century,” and has received increasing attention in the biological sciences field (Zhao et al., 2018; Zhang et al., 2021). THz waves refer to electromagnetic waves with a frequency between 0.1 and 10 THz and a position between microwave and infrared radiation. Under THz radiation, the chemical bonds of the molecules of various nutrients are broken and formed within picoseconds, resulting in the strong absorption of THz waves. Thus, THz spectroscopy can be used for the detection of the nitrogen content of crops. While THz spectroscopy has been widely used, due to the limitations of detection objects and technical means, its application in the field of agricultural engineering remains in its infancy. Some scholars have found that the spectral resolution of THz time-domain spectroscopy (THz-TDS) can be used to identify the composition of objects, and THz imaging technology can be used to identify nutrients such as chlorophyll, lutein, and the nitrogen-to-sugar ratio. Characteristic fingerprints lacking internal components and macromolecules can be used to diagnose the internal structure of crops with different nutrients (Liu et al., 2020; Zhang et al., 2022). For example, Liu et al. (Liu and Han, 2014) took advantage of the fact that the absorption of the THz spectra of proteins, amino acids, and other substances in biscuits is much less than that of water. They conducted respective model analyses on the frequency domain, refractive index, and absorption coefficient of THz spectral data, and obtained the best effect of the absorption coefficient model. Long et al. (2017) used a THz spectrometer to obtain the spectral data of leaves *in vitro* in a point-by-point scanning manner, and observed the differences of different water contents under image reconstruction. The regression prediction model was established according to the mean values in the time and frequency domains and the measured water content of the THz image of the leaf. These previous studies prove the feasibility of using THz spectroscopy to detect crop nutrition.

Therefore, in view of the current shortcomings, the advantages of THz imaging technology were used in this research for the identification of chlorophyll, lutein, the nitrogen-to-sugar ratio, and other nutritionally abundant internal components and characteristic fingerprints of macromolecules. The detection accuracy of the nitrogen content of tomato leaves is expected to be improved by using the algorithm to detect crop nutrition.

## 2 Materials and methods

### 2.1 Sample cultivation

**Figure 1:**
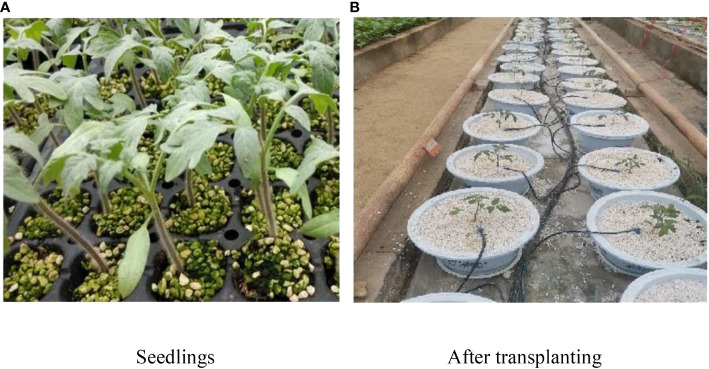
The sample cultivation. **(A)** Before smoothing **(B)** After smoothing.

The quality of the test sample cultivation has a direct impact on the test results. Therefore, in the process of sample cultivation, the influence of environmental factors should be minimized and the accuracy of the sample data should be improved. The experiment was carried out in a Venlo-type greenhouse (32.2°N, 119.5°E) at the Key Laboratory of Modern Agricultural Equipment and Technology, Ministry of Education, Jiangsu University. The environmental temperature of the greenhouse was maintained at 10.7-29.4°C, and the relative humidity was 37.3%-87.9%. The test samples were 906 red tomatoes (Shanghai Changchong Tomato Seed Industry Co., Ltd.). Tomato seeds with large, plump grains and similar shapes were selected, and the selected seeds were placed in lightly salted water to screen out diseased seeds and sclerotia. Additionally, a 0.3 m × 0.6 m black plastic plug tray was selected as the seedling-raising device. The seedling base was composed of vermiculite, perlite, and peat at a ratio of 1:3:1. The screened seeds were evenly sown in the plastic plug tray. After the seedlings had three true leaves, they were transplanted into a plastic round pot with a radius of 10.5 cm and a height of 29 cm. To achieve the purpose of soilless cultivation, perlite with strong root fixation was selected as the matrix. Each sample was repeated 30 times. During the experiment, the cultured tomato plants were watered with the Japanese Yamazaki nutrient solution formula (Mao et al., 2022). **Figure 1** displays the sample cultivation and transplanting site.

### 2.2 Equipment

**Figure 2:**
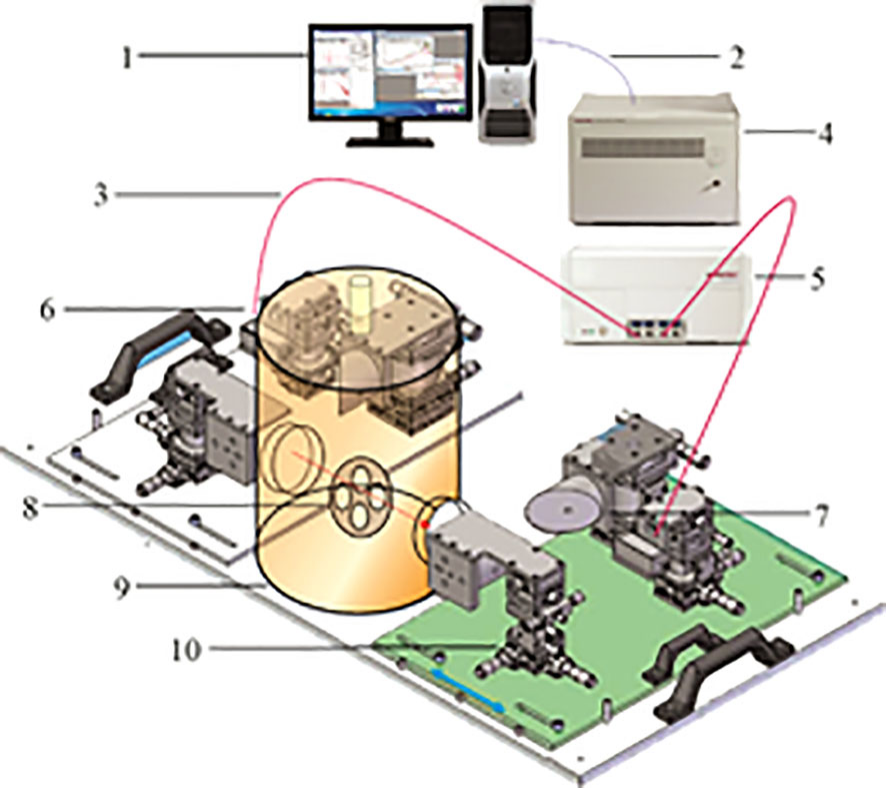
The structure and working principle of the Advantest-TS7400 THz-TDS measurement system.

The TS7400 THz–TDS measurement system produced by Japan’s ADVAN Corporation was used to collect the THz information of the samples. The system is specially customized for the detection of agricultural biological information. It has an attenuated total reflection (ATR) module and can perceive high water contents for the detection of biological tissue and living samples. **Figure 2** shows the structure of the TS7400 THz-TDS measurement system.

1. Operating/analyzing computers; 2. Ethernet; 3. Optical fiber; 4. Analysis unit; 5. Measurement unit; 6. THz transmitter; 7. THz detector; 8. Sample stage; 9. Cryostat transfer module; 10. Removable stand.

The working principle of the TS7400 THz-TDS measurement system is as follows. The THz measurement unit, the THz transmitter, and the THz detector are connected by optical fiber without adjusting the external optical path. The THz transmitter emits laser pulses that are divided into two mutually perpendicular beams under the action of the beam splitter. One laser beam is a stronger pump light, and the other is a weaker probe light. The pump light is incident on the emitting crystal to generate a THz pulse that passes through the sample stage through the mirror. It is then transmitted to the THz detector through the detection crystal collinear with the probe light that has undergone multiple reflections. The value is transmitted to the control computer. After the control computer receives the signal, the analysis unit can directly calculate parameters such as the refractive index, absorption coefficient, and dielectric constant of the sample, and the time-domain THz spectrum and distribution information of the sample can be obtained. Compared with the traditional THz device, this device not only has higher accuracy, but the size of the detectable sample is also expanded from a maximum of 3 cm² to 225 cm², which can better meet the measurement needs of crop samples.

### 2.3 Sample data collection and processing

#### 2.3.1 Sample data collection

Samples were collected from the tomato plants after 65 days of nitrogen stress treatment. During leaf collection, the healthy 7-leaf pinnate compound leaves of the tomato that best reflect the growth state were selected and cut off. They were immediately placed in a sealed bag to maintain freshness, which was placed in a portable refrigerated incubator (Ni et al., 2021) to prevent the external environment from affecting it. Twenty leaf samples were selected for each nitrogen stress gradient, and a total of 80 samples were collected from four gradients. The samples were then placed in the THz-TDS measurement system for sample scanning to obtain the spectral information. Before the experiment, a dehumidifier was turned on, and the relative humidity in the sample detection box was reduced to below 5% to eliminate the interference of water vapor on the THz spectrum. Ten sampling points were scanned for each sample to obtain the spectral information, and the average value was taken as the data collected for the sample.

**Figure 3:**
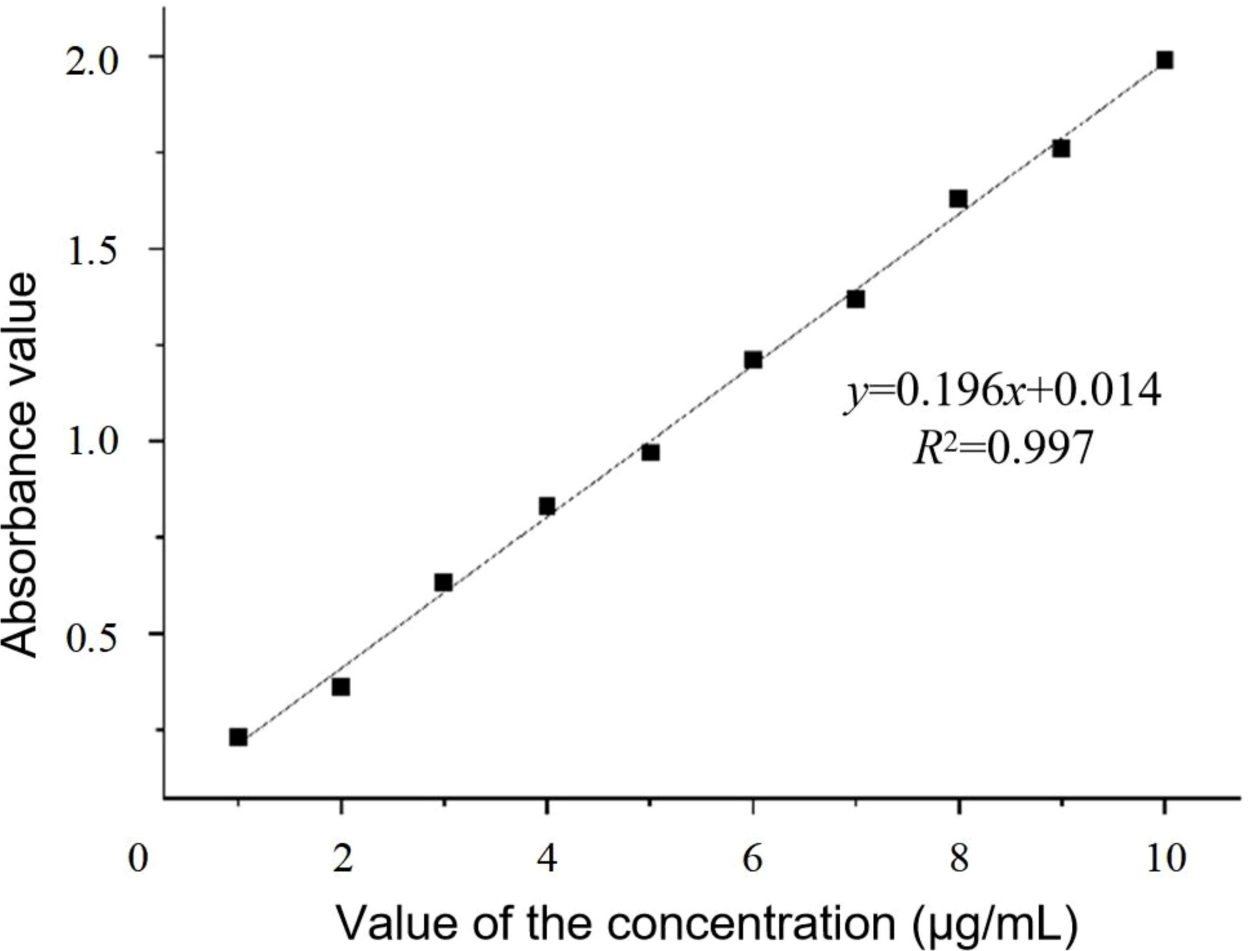
The standard curve of the nitrogen content.

The nitrogen content of the collected test samples was determined by the Nessler reagent colorimetric method, and the colorimetry was performed with a spectrophotometer at 420 nm. The actual measurement value of the nitrogen content of the sample was calculated by drawing the standard curve of the nitrogen content concentration and the photometric values, as presented in **Figure 3**.

#### 2.3.2 Data smoothing

**Figure 4:**
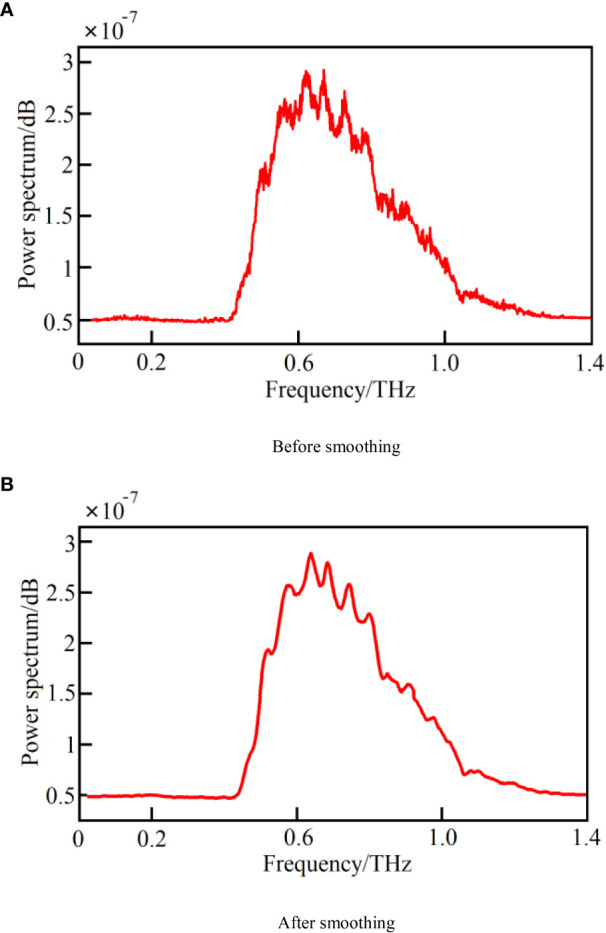
The comparison of the THz power spectrum before and after smoothing. **(A)** Before smoothing, **(B)** After smoothing.

The Savitzky-Golay (S-G) smoothing algorithm is a commonly used algorithm in data preprocessing due to its simple, fast, and easy-to-use process. The principle is to first take a window with an odd number of points in width, and to use the least-squares method for fitting *via* the translation of the window. The original value is then replaced with the fitted value of the midpoint of the window to achieve the smoothing of the data (Zhao et al., 2018). In this study, the S-G smoothing algorithm was used to preprocess the data, and the window width was 7 points/time. Taking the power spectrum data as an example, the comparison of the effect before and after the smoothing of the THz power spectrum is shown in **Figure 4**. The results demonstrate that the algorithm can effectively reduce the interference signal and improve the modeling efficiency and model accuracy.

#### 2.3.3 Data set partitioning

The division of the sample set is the key to the applicability of the model. If the selected calibration set has good representativeness, the predictive ability of the model can be enhanced.

The random sampling (RS) algorithm is a simple method of randomly extracting samples regularly or irregularly from the entire sample set. A portion of the samples can be randomly selected as the prediction set until the sampling is full, and the remaining portion is used as the calibration set. The Kennard-Stone (KS) method was jointly proposed by Kennard and Stone (He et al., 2018), and its sample screening process of the calibration set is as follows. By calculating the Euclidean distance between two samples, the two samples with the largest distance enter the calibration set. The distance between the two selected samples is selected, the shortest distance among them is selected, and the sample corresponding to the longest distance among these shortest distances is entered into the calibration set. This method can ensure that the calibration set samples are evenly distributed according to the spatial distance, and the Euclidean distance between the two vectors is calculated as follows:


(1) $$d_{x}(p,q)=\sqrt{\sum_{j-1}^{J}{\left[x_{p}(j)-x_{q}(j)\right]}^{2}}$$

where *d*_*x*_(*p*,*q*) is the Euclidean distance of the spectral reflectance between samples *p* and *q*, is the reflectance of sample *p* at the *j*^th^ wavelength point, *x*_*q*_(*j*) is the reflectance of *q* at the *j*^th^ wavelength the reflectivity of each wavelength point, and *J* is the number of wavelength points.

**Table 1** reports the results of the power spectrum and absorbance after sample division by the RS and KS algorithms.

**Table 1:** The results of sample division by the RS and KS algorithms.

Method	*R*^2^	RMSE
RS	0.8427	0.1379
KS	0.8922	0.1003

The data in the calibration set obtained by sample division by the KS algorithm had a higher determination coefficient and a lower root-mean-square error (RMSE) than the data obtained by the RS algorithm. The subsequent data analysis and processing were therefore carried out on the basis of the division of the sample data set by the KS algorithm.

### 2.4 Model construction method

#### 2.4.1 Uninformative variable elimination

Uninformative variable elimination (UVE) (Jiang et al., 2019) can filter out spectral variables that contribute less to the modeling, which allows for the selection of representative spectral variables. The filtered variables are called uninformative variables, and by filtering them out, the complexity of the model and the number of variables required for subsequent modeling can be reduced. The UVE algorithm is based on the partial least-squares (PLS) algorithm. During variable screening, artificial noise variables equal to the original variables are added to the PLS model. The variables are randomly numbered, and one is left by crossover. This method is used to obtain regression coefficients of variables including artificial noise. In the analysis, the random change generated by artificial noise is used as a reference, and the reliability of each variable is measured by the threshold and the stability value. When the absolute value of the stability is less than the threshold, the variables in this part are regarded as non-informative variables. The stability value is defined as follows:


(2) $$S_{i}=\frac{mean(b_{i})}{std(b_{i})};\;i=1,2,3\cdots ,m$$

where *i* is the sample variable number, *S*_i_ is the stability value of the variable numbered *i*, *b_i_* is the regression coefficient of the variable numbered *i*, *std*(*b_i_*) is the regression coefficient of *b_i_*, *mean*(*b_i_*) is the mean value of *b_i_*, and *m* is the total number of variables.

The steps of using the UVE algorithm to filter the feature frequencies are as follows.

(1) A *λ×µ* noise matrix is artificially generated, the spectral matrix is set as *X*, and the spectral matrix and noise matrix are spliced into a new matrix *P* of *λ*×2*µ*.
(2) Using the cross leave-one-out method, a regression analysis is performed on the matrix *P*, and the regression coefficient matrix *L* (*λ*×2*µ*) is obtained.
(3) The mean value *mean*(*b_i_*) and the regression coefficient *std*(*b_i_*) are respectively calculated, and the stability value matrix corresponding to each variable is obtained by Eq. (4).
(4) In the range of the noise variable interval [*µ*+1, 2*µ*], the maximum and minimum values of the stability value matrix are obtained, and the characteristic variable is selected in the range of [1, *µ*].

#### 2.4.2 Stability competition adaptive reweighting sampling algorithm

Stability competitive adaptive reweighted sampling (SCARS) (Liu et al., 2014) is a common algorithm used to screen the optimal feature combination. During calculation, the measured THz spectral data can be set as a matrix *X_N×P_*. The number of samples is *N*, *P* is the number of variables, and the specific operation steps of SCARS are as follows.

(1) The stability value *c_j_* of each frequency-band variable is calculated as follows:


, (3) $$c_{j}=\left|\frac{b_{j}}{s(b_{j})}\right|;j=1,2,\cdots P$$

where *c_j_* is the stability value of the *j*^th^ variable during Monte Carlo (M) sampling, *b_j_* is the value of the *j*^th^ variable during M sampling, and *s(b_j_)* is the standard deviation of the *j*^th^ variable during M sampling.

(2) The adaptive reweighted sampling method is combined with forced frequency band selection to screen groups with large stability values. They are combined into a subset of variables, and the ratio of variables to the whole frequency band is determined by the exponential decay function (EDF).

(3) These two steps are repeated in turn to obtain variable subset *K*. A PLS regression (PLSR) model is obtained, and the obtained variable subset is then evaluated through tenfold cross-validation. The *K* value is the number of operations in the SCARS algorithm, and the RMSE of cross-validation (RMSECV) value can be used as the judgment basis for whether the variable subset is a feature variable subset. The feature variable subset can be obtained at the smallest value of the RMSECV.

#### 2.4.3 Interval partial least-squares algorithm

Interval PLS (iPLS) (De Lima et al., 2012) is a commonly used interval filtering algorithm for characteristic variables that was proposed by Norgaard at the beginning of the 20^th^ century. Based on the PLSR model, the algorithm divides the overall intervals into equal intervals to be filtered. The PLSR model of each equally spaced interval *n* is respectively established. By comparing the model accuracy of each subinterval, the subinterval with the best accuracy is selected as the modeling candidate frequency interval.

#### 2.4.4 Radial basis function neural network

Radial basis function neural networks (RBFNNs) are developed based on the multi-dimensional spatial interpolation of radial basis functions. They are feed-forward neural networks with good performance and can be understood as function approximation or curve fitting in high-dimensional space. The proposal of the RBFNN provided new ideas and methods for the application and research of neural networks in various fields. In practical applications, this neural network has the advantages of a fast learning speed, no local minimum problem, and the ability to establish a corresponding network topology according to different types of data (Gao et al., 2014).

The RBFNN is mainly composed of an input layer, a hidden layer, and an output layer. When using this neural network to establish the model, *P* can be set as the sample input matrix of the correction set, and *T* is the sample output matrix of the correction set. The calculation formula of hidden-layer neurons can be obtained as follows:


, (4) $$a_{i}=\exp(-{\left\|C-p_{i}\right\|}^{2}b_{i});i=1,2,\cdots ,Q$$

where *Q* is the number of calibration set samples, and *C* is the center of the radial basis function. The connection weight between the hidden layer and input layer is set as *W*. Moreover, *b_2_* is the threshold value of *N* output layer neurons, and the following formula can be obtained.


(5) $$\left[W;b_{2}\right]\times \left[A;I\right]=T;A=\left[a1,a2,\cdots ,a_{Q}\right];I={\left[1,1,\cdots ,1\right]}_{1\times Q}$$

By solving Eq. (5), the threshold value *b_2_* and the connection weight value *W* between the output layer and the hidden layer can be obtained as follows.


(6) $$\left\{ \begin{array}{l} Wb=T/\left[A;I\right]\\ W=Wb\left(:,1:Q\right)\\ b_{2}=Wb\left(;,Q+1\right) \end{array}\right.$$

#### 2.4.5 Backpropagation neural network

Backpropagation neural networks (BPNNs) (Li et al., 2020; Liu et al., 2021) can achieve the goal of not solving the relevant mapping equation in advance by learning the relationship between the input and output. Because of its high fault tolerance and parallel processing ability, the BPNN has a wide range of applications in target classification, recognition, and optimization.

The BPNN algorithm is mainly composed of forward calculation and error-reverse calculation. The topology includes an input layer, a hidden layer, and an output layer. During forward calculation, when the signal received by the output layer is an unexpected output value, a reverse propagation error signal will be generated, and the propagation path is the initial connection path. Compared with the RBFNN, the BPNN has the following characteristics.

(1) It has more hidden layers and hidden-layer nodes, and it can approximate any nonlinear mapping relationship.
(2) BPNNs are global approximation algorithms, which improves their generalization ability at the cost of reducing model accuracy.
(3) The ownership value must be updated during each sample learning. While this increases the robustness of the reverse neural network model, the update of the weight value slows down the convergence speed and the model easily falls into the local minimum.

## 3 Results and discussion

To build the model for the prediction of the nitrogen content of tomato, all samples were divided into calibration and prediction sets. There were 80 samples to be tested, of which 60 were included in the calibration set and 20 were included in the prediction set. The RMSE was used to evaluate the fitting accuracy of the calibration set model, and the coefficient of determination (*R*^2^) was used to examine the degree of correlation. Their calculation formulas are as follows:


(7) $$RMSE=\sqrt{\frac{\sum_{i=1}^{n}(y-\hat{y}{)}^{2}}{n-1}}$$

(8) $$R^{2}=\frac{\sum {\left(\hat{y}-\bar{y}\right)}^{2}}{\sum {\left(\hat{y}-\bar{y}\right)}^{2}}=1-\frac{\sum {\left(y-\hat{y}\right)}^{2}}{\sum {\left(y-\bar{y}\right)}^{2}}$$

where *y* is the measured value of the *i*^th^ sample in the calibration set, *ŷ* is the predicted value of the *i*^th^ sample in the calibration set, and *ȳ* is the average value of the measured values of all samples in the calibration set.

### 3.1 Extraction of THz spectral information from tomato leaves

**Figure 5:**
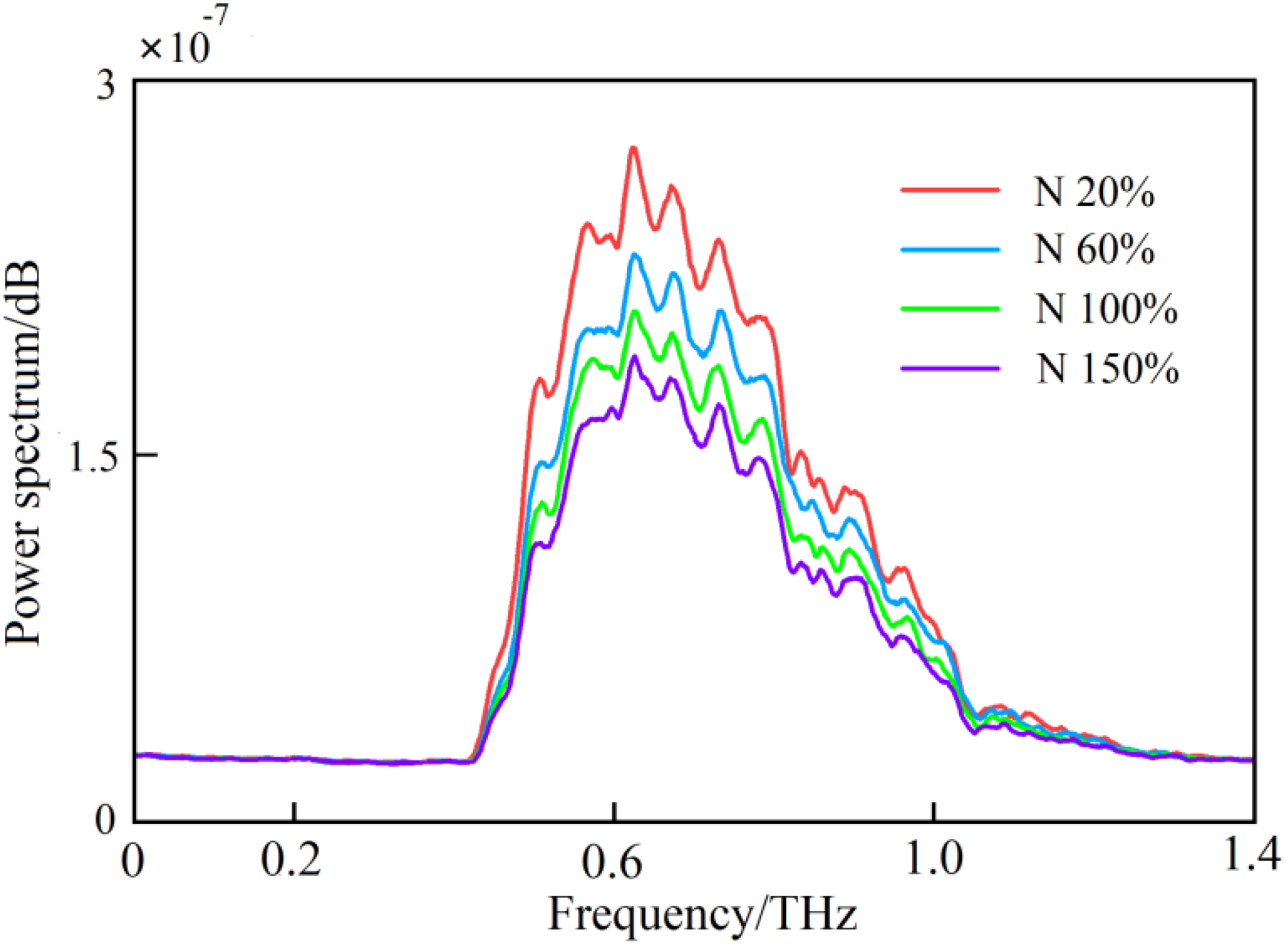
The THz frequency-domain spectra of leaf samples with different nitrogen contents.

In the frequency-domain spectrum, with the change of the band, the samples have different absorptions of THz waves at different frequencies, as shown in **Figure 5**. The figure presents the THz frequency-domain spectral curves of four different nitrogen-stressed tomato leaf samples, from which it is evident that the trends of various spectral curves were roughly similar.

By preprocessing the raw THz spectral data, the vast majority of invalid information and noise signals in the data were removed. It can also be seen from **Figure 5** that there were obvious gradient differences in the THz power spectrum curves of the samples under different nitrogen stress gradients. In the power spectrum graph, the value of the power spectrum shows a trend of first increasing and then decreasing, and it reached a peak value of around 0.7 THz. At the peak value and its surrounding frequencies, the nitrogen stress gradient curves presented obvious stratification. However, when the frequency was too small or too large, the THz power spectrum of each stress gradient was too dense, and the discrimination was not obvious.

#### 3.1.1 Processing results of the uninformative variable removal algorithm

**Figure 6:**
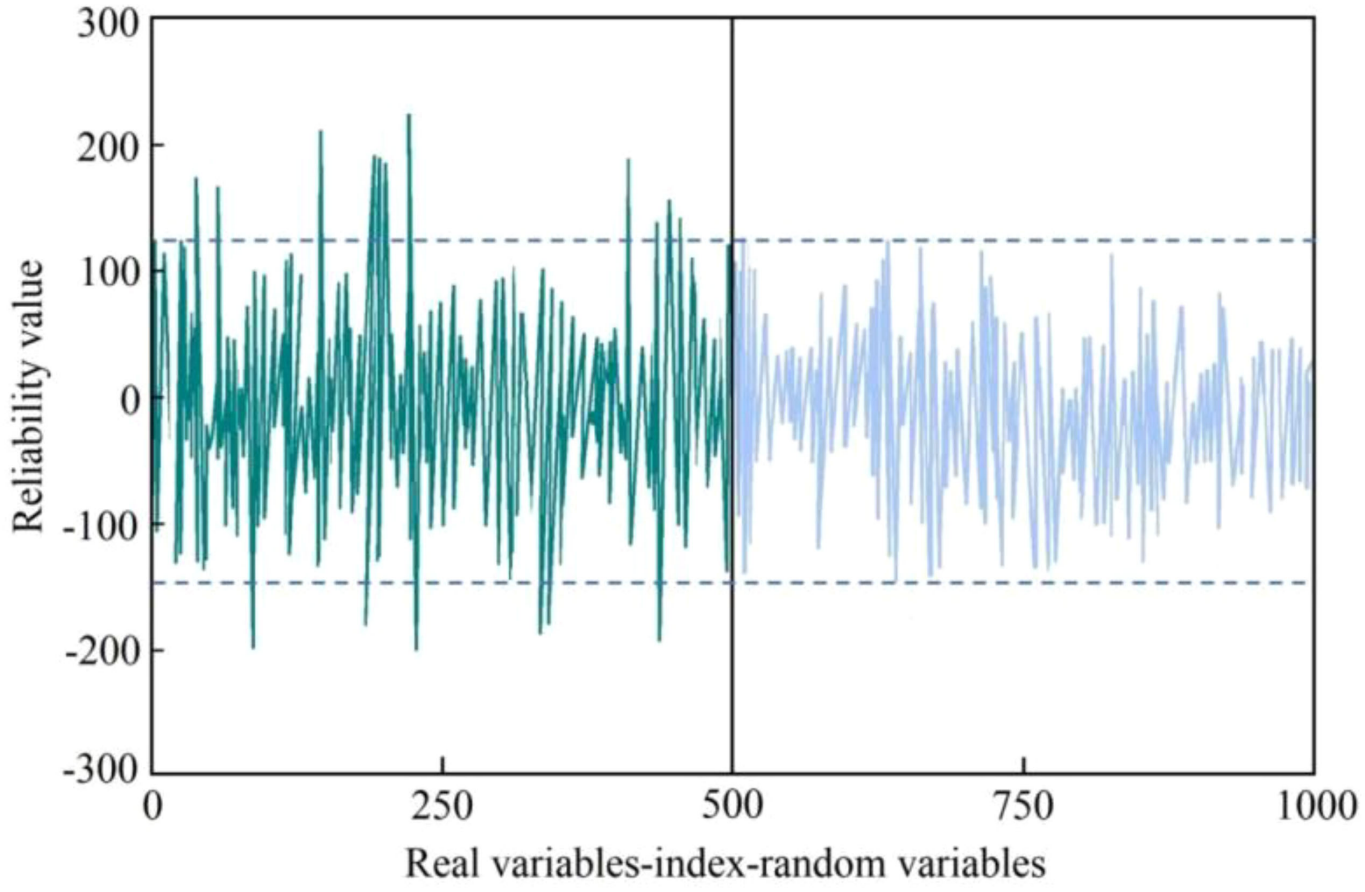
The UVE feature frequency point screening results.

**Figure 6** shows the results of the uninformative variable screening.

**Figure 7:**
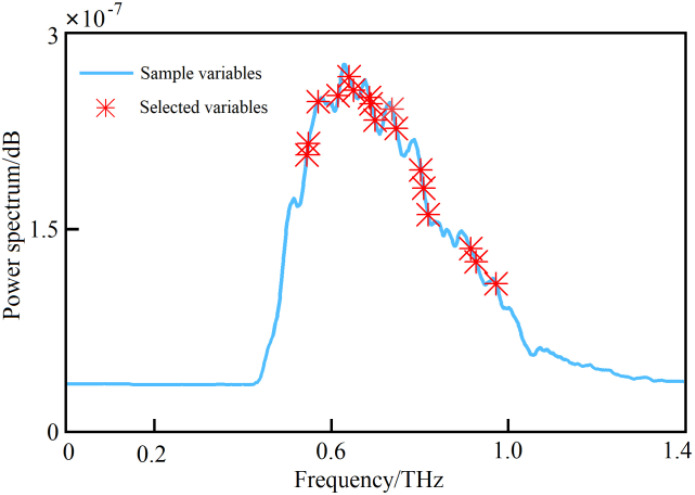
The characteristic bands selected based on the UVE algorithm.

**Figure 8:**
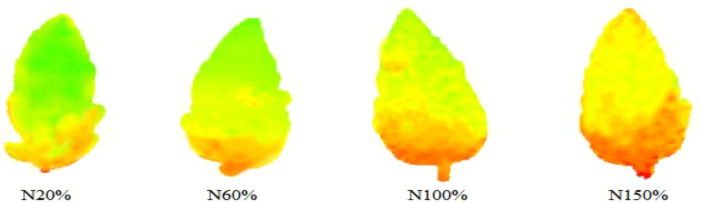
THz images of leaves with different nitrogen levels in the characteristic frequency bands screened by the UVE algorithm.

In **Figure 6**, the left side is the THz spectrum variable, the right side is the artificially generated noise variable, and the abscissa and the ordinate are respectively the serial number and stability index corresponding to the variable. The larger the absolute value of the stability index corresponding to the variable, the greater the correlation with the model. The dotted line in the figure is the screening threshold set based on artificial noise. Based on this threshold, a total of 17 characteristic frequency bands were screened, as shown in **Figures 7**, **8**.

#### 3.1.2 Processing results for the stability competition adaptive reweighting sampling algorithm

**Figure 9:**
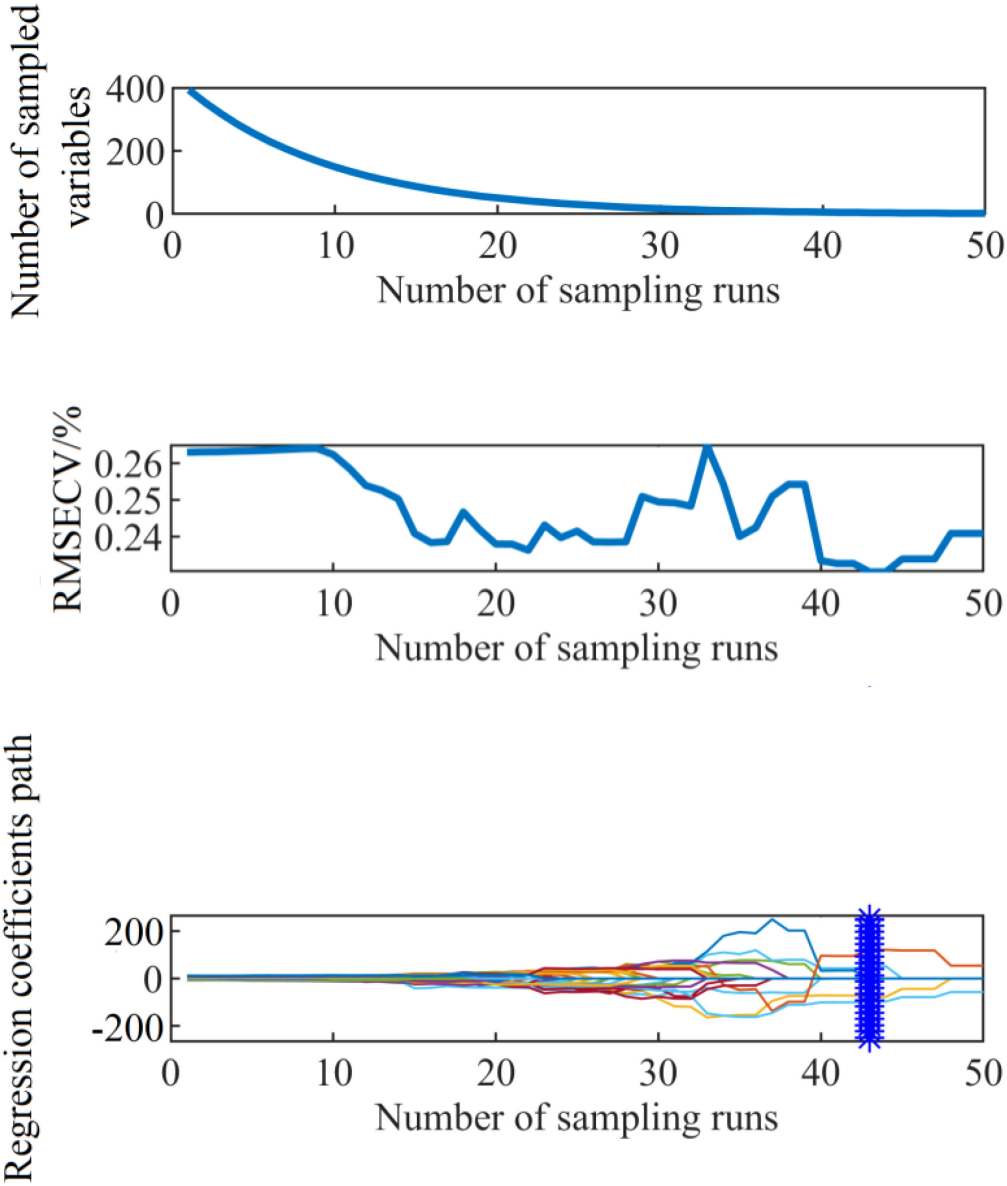
The running process of the SCARS algorithm.

In this study, when using the SCARS algorithm for filtering, the number of cycle sampling instances was 50 and THz power spectrum data were taken as the object. After 50 rounds of sampling had been reached, each index value had reached a stable state. The processing result of the SCARS algorithm is shown in **Figure 9**.

**Figure 10:**
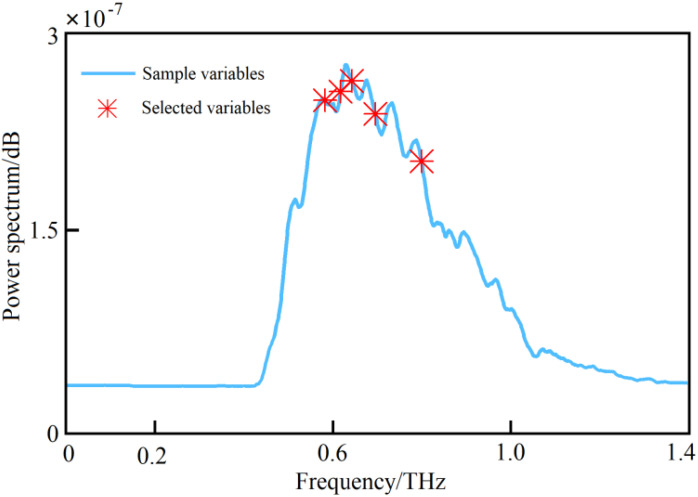
The characteristic bands selected based on the SCARS algorithm.

**Figure 11:**
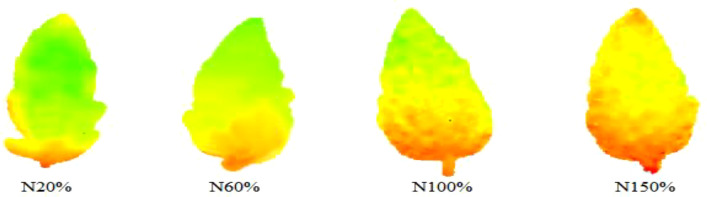
THz images of leaves with different nitrogen levels in the characteristic frequency bands screened by the SCARS algorithm.

It can be seen from **Figure 9** that with the increase of the number of iterations of the SCARS algorithm, the number of frequency bands retained generally decreased. However, the speed of reduction slowed down, indicating that the SCARS algorithm used coarse screening in the early stages of screening the characteristic frequency bands and fine screening in the later stages. It can be seen that with the gradual increase of the number of runs, the RMSECV value presented a decreasing trend. The RMSECV value was the minimum when the number of runs reached 43, which indicates that the frequency band with less correlation with the sample nitrogen content had been removed before. After 43 runs, the value had rebounded, and, combined with the change in the regression coefficient path, this indicates that the characteristic frequency bands related to the sample nitrogen content may have been removed by mistake. When the RMSECV value was the lowest, the subset of selected characteristic frequency bands was the best. Five characteristic frequencies were obtained, namely 0.574, 0.624, 0.642, 0.704, and 0.817 THz, and they were used as alternative characteristic frequency bands. The selected THz spectral bands are shown in **Figures 10**, **11**.

#### 3.1.3 Processing results for the interval partial least squares algorithm

**Figure 12:**
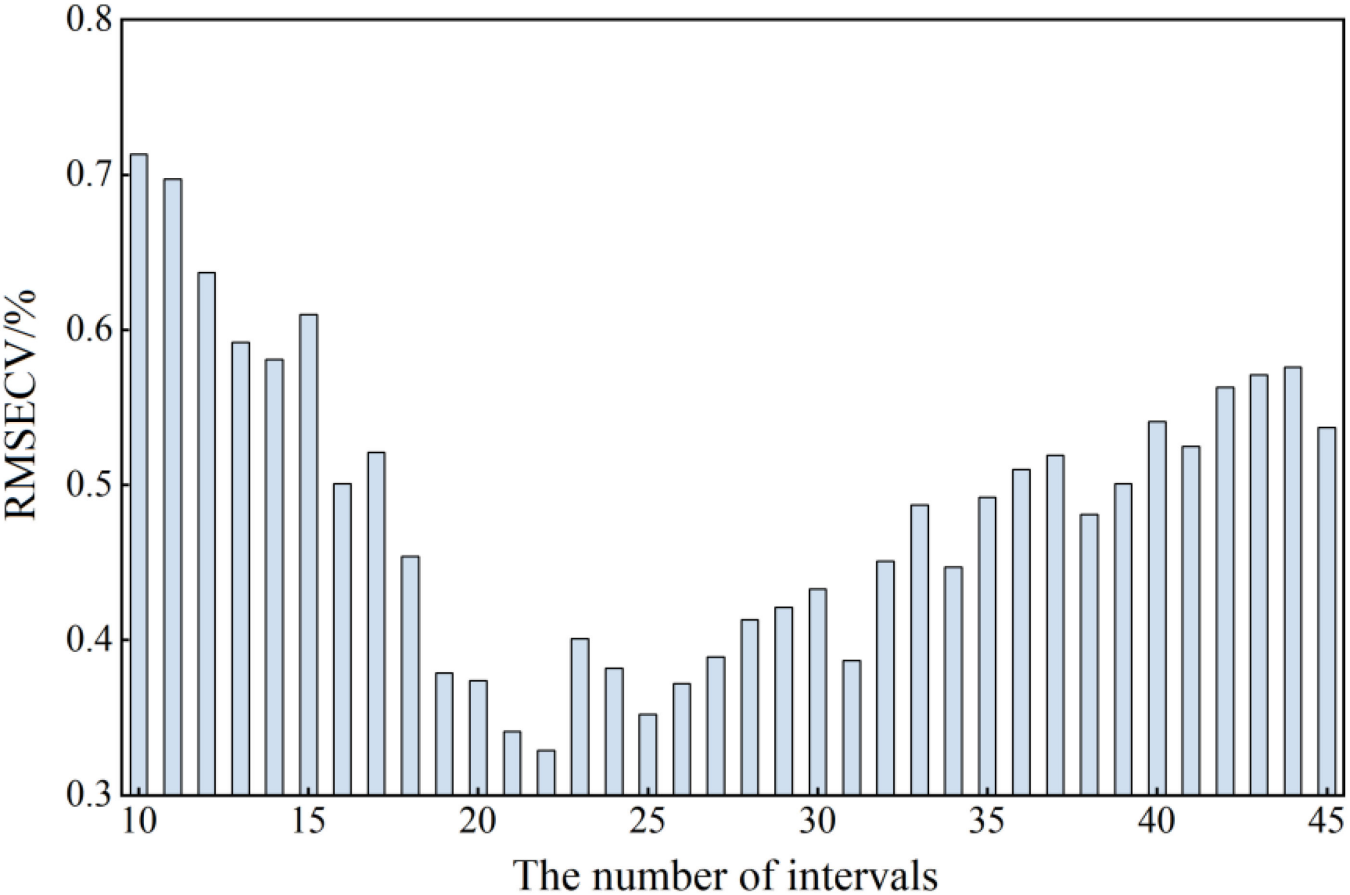
The results of the optimal number of isometric interval partitions.

When using the iPLS algorithm to filter the optimal frequency band interval, the number of subintervals to be divided must first be determined. Too many or too few subintervals will affect the subsequent selection of the optimal frequency band interval. In this study, the pretreated THz spectral intervals were divided into 10-45 equidistant intervals. As shown in **Figure 12**, when the RMSECV value was the minimum, the overall THz spectral interval was divided into 22 equidistant subintervals.

**Figure 13:**
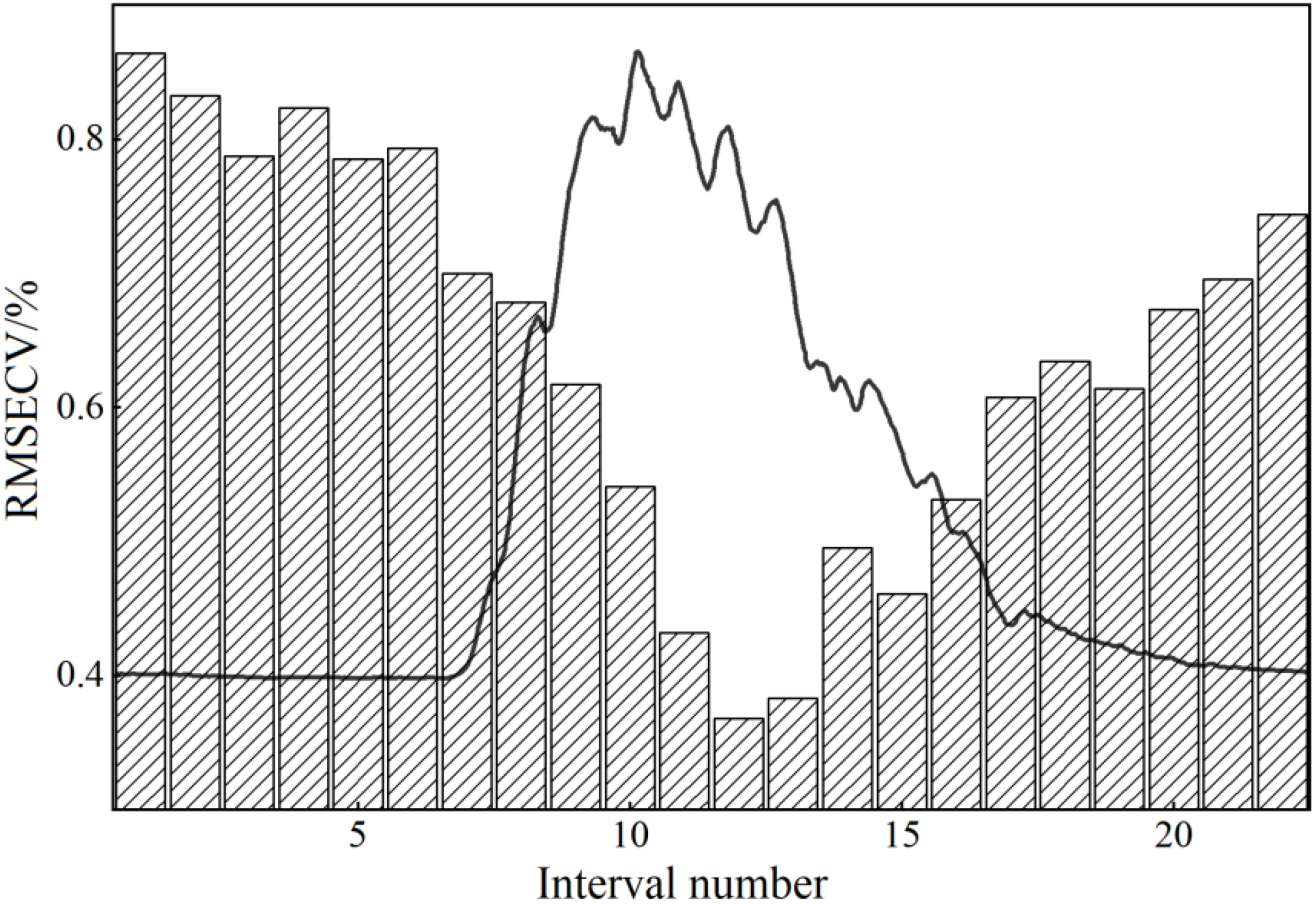
The results of running the iPLS algorithm.

**Figure 14:**
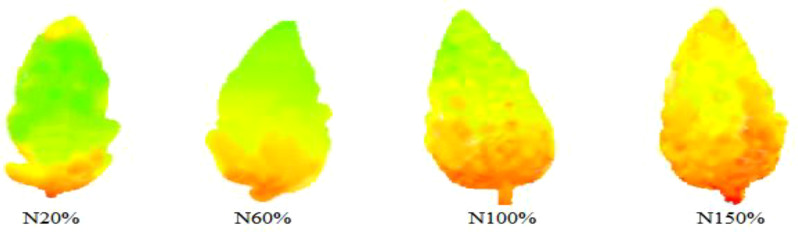
THz images of leaves with different nitrogen levels in the characteristic frequency bands screened by the iPLS algorithm.

According to the number of subintervals shown in **Figure 12**, the THz spectral interval in the range of 0-1.4 THz was divided into 22 equal parts, and the PLS model index values in each subinterval were calculated. **Table 2** reports the THz spectral frequency division range of each subinterval and the corresponding RMSECV value. **Figures 13**, **14** show the operation results of the iPLS algorithm.

**Table 2:** The index values of each subinterval model.

Number	Frequency range (THz)	RMSECV (%)	Number	Frequency range (THz)	RMSECV (%)
1	0-0.0636	0.8641	12	0.6996-0.7632	0.3671
2	0.0636 ~0.1272	0.8327	13	0.7632-0.8268	0.3826
3	0.1272-0.1908	0.7874	14	0.8268-0.8904	0.4952
4	0.1908-0.2544	0.8234	15	0.8904-0.9540	0.4611
5	0.2544-0.3180	0.7851	16	0.9540-1.0176	0.5312
6	0.3180-0.3816	0.7687	17	1.0176-1.0812	0.6074
7	0.3816-0.4452	0.7002	18	1.0812-1.1448	0.6341
8	0.4452-0.5088	0.6784	19	1.1448-1.2084	0.6139
9	0.5088-0.5724	0.6174	20	1.2084-1.2720	0.6732
10	0.5724-0.6360	0.5412	21	1.2720-1.3356	0.6954
11	0.6360-0.6996	0.4317	22	1.3356-1.4000	0.7441

It can be seen from **Figure 13** that the index values of the PLS model established in each subinterval presented a trend of first decreasing and then increasing, and were closely related to the power spectrum curves of each stress gradient. When the power spectrum curve density was large, the corresponding modeling effect was poor; on the contrary, the modeling effect was better. Finally, the subinterval with the interval number of 12 was selected as the characteristic frequency subinterval, and there were 31 characteristic frequency points in total.

### 3.2 Establishment and analysis of the model

#### 3.2.1 Results for the radial basis function neural network

**Figure 15:**
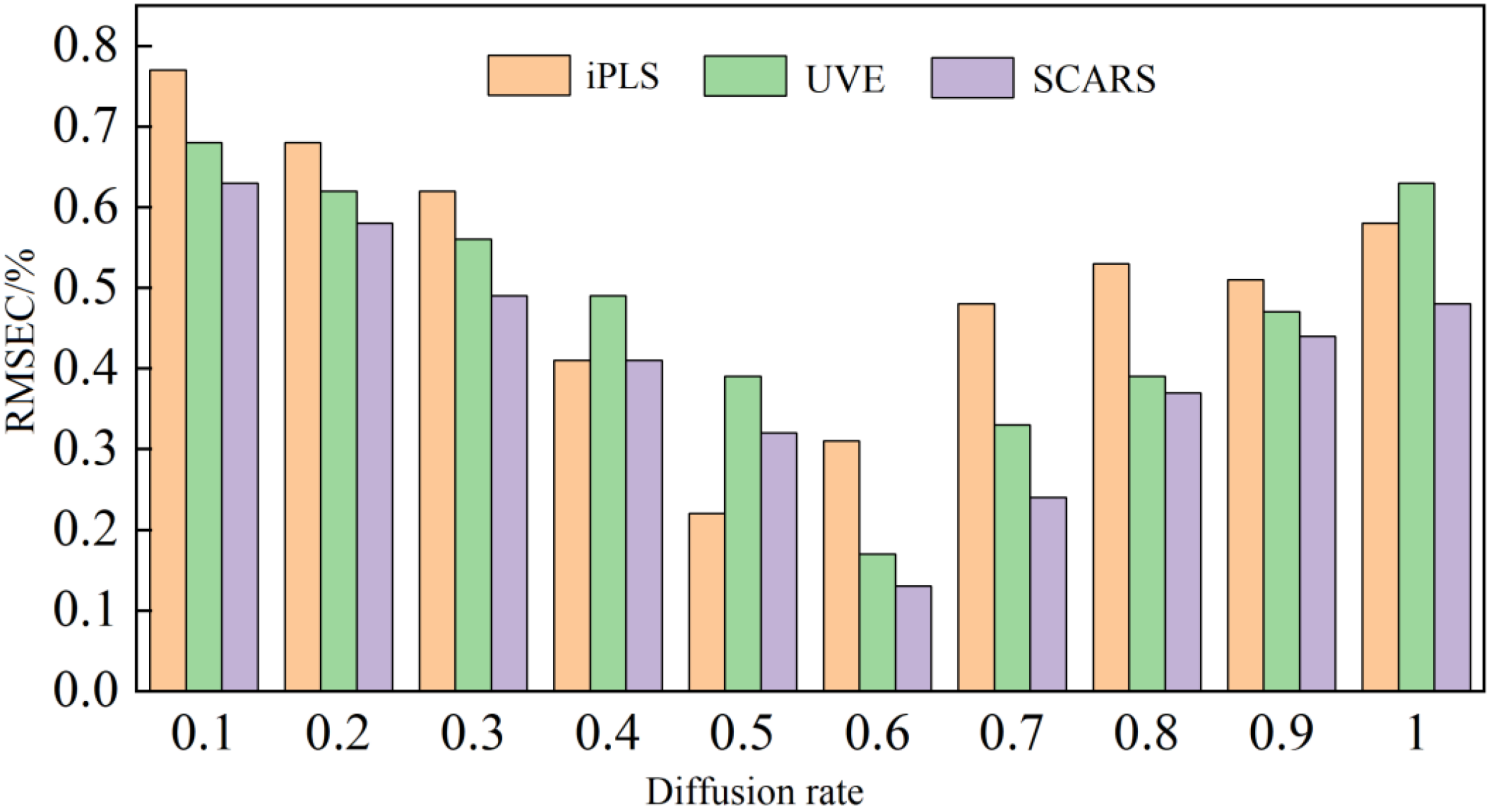
The influence of the diffusion velocity on the RBFNN model.

An RBFNN with strict logic was established by using the function package in MATLAB software. The expansion speed is the key to determining the quality of the RBFNN, and the setting of this parameter depends on the target object. **Figure 15** exhibits the change of the RMSE of correction (RMSEC) of the RBFNN, iPLS, UVE, and SCARS models with an expansion speed of 0.1-1 and an interval of 0.1.

It can be seen from **Figure 15** that when the diffusion speed was 0.5 and 0.6, the RBFNN model had the best RMSEC value. The established neural network model was tested to verify the prediction set, and the prediction results are reported in **Table 3**.

**Table 3:** The prediction results of the RBFNN model based on the THz spectrum.

Algorithm for band screening	RMSEC (%)	*R*_c_^2^	RMSEP (%)	*R*_p_^2^
UVE	0.1721	0.8457	0.2197	0.8218
SCARS	0.1322	0.8714	0.1855	0.8463
iPLS	0.2214	0.8352	0.2784	0.8149

**Figure 16:**
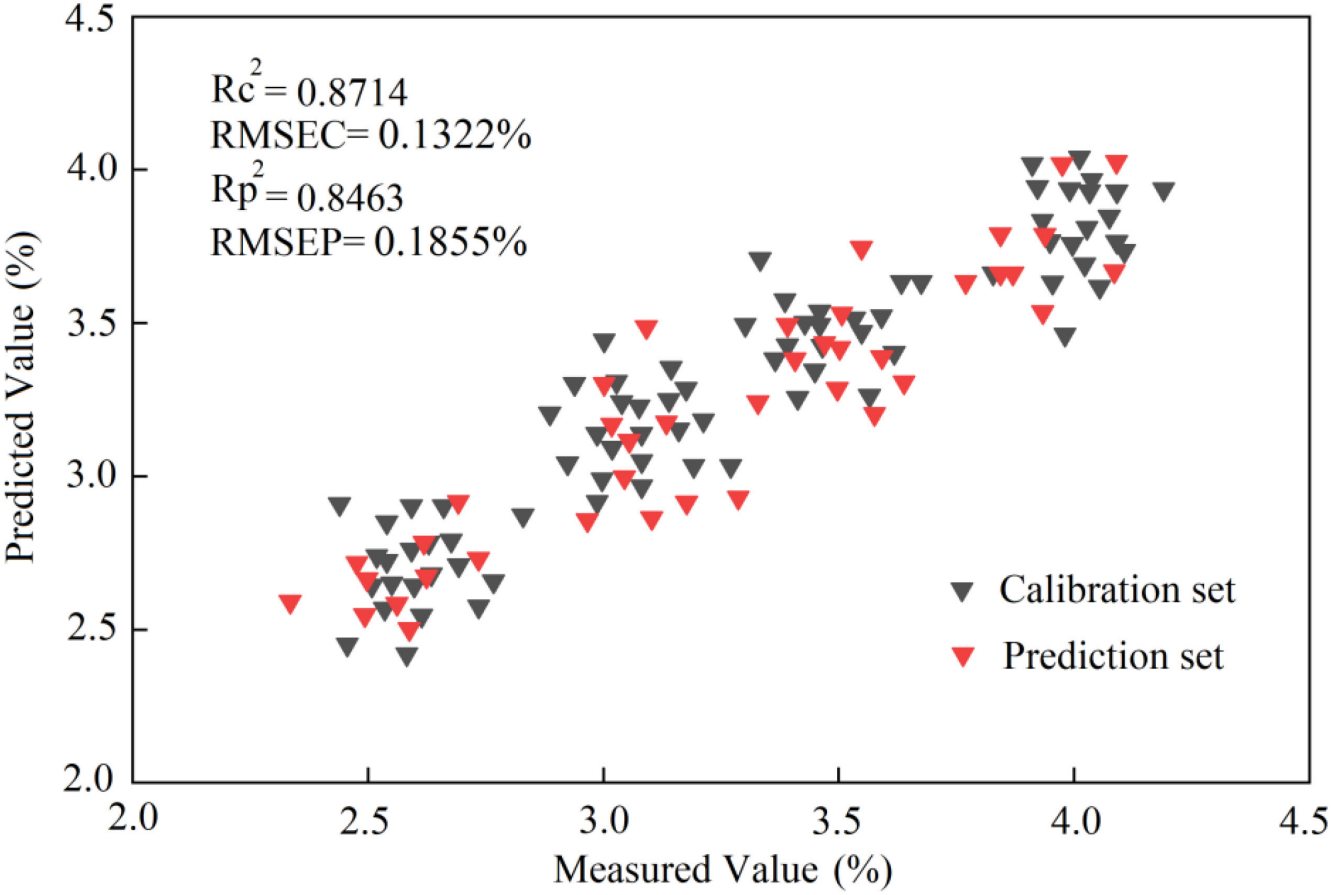
The scatter plot of the prediction results of the RBFNN model based on THz spectroscopy.

According to the results reported in **Table 3**, when the RBFNN algorithm was combined with the characteristic bands screened by SCARS, the detection model had the best effect. The RMSEC and the RMSE of prediction (RMSEP) were respectively 0.1322% and 0.1855%, and the determination coefficients of the correction set (*R*_c_^2^) and prediction set (*R*_p_^2^) were respectively 0.8714 and 0.8463. The scatter plot of the optimal results of the model based on the RBFNN algorithm is presented in **Figure 16**.

#### 3.2.2 Results for the backpropagation neural network

**Figure 17:**
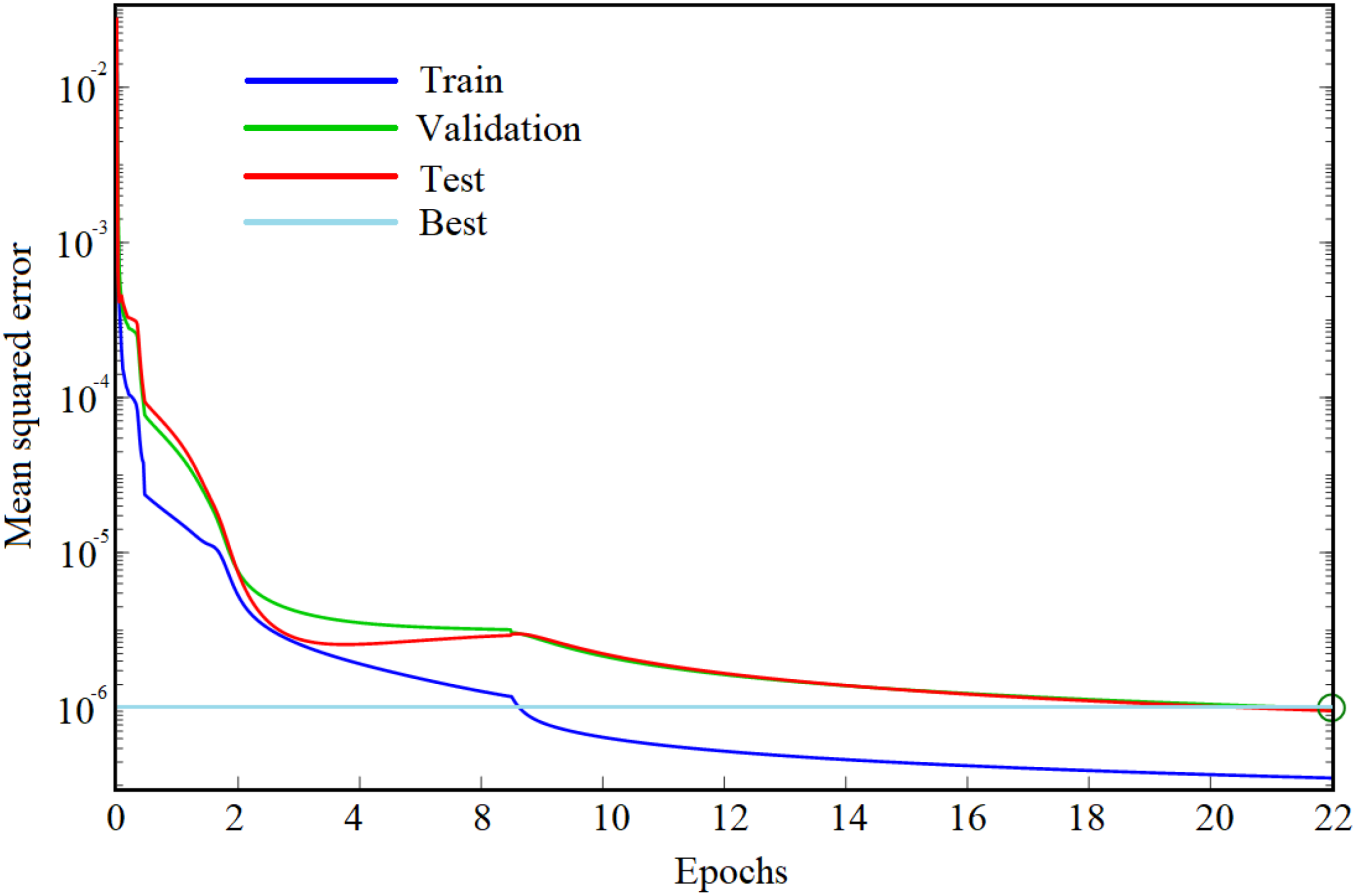
The training process of the BPNN model.

The sample data were trained using the scaled conjugate gradient function in the BPNN tool in MATLAB software. The specific training process included using the newff function to construct the basic structure of the BPNN. Moreover, the architecture based on the BPNN trained the model through the Trainlm function, and the Sim function output model was used to train the process. Taking the combination of characteristic bands screened by the iPLS algorithm as an example, the training process of its BPNN is shown in **Figure 17**.

It can be seen from **Figure 17** that the number of network iterations epochs was 22. Moreover, the learning rate was set to 0.01, and the number of hidden-layer neurons was set to 6. The sample data filtered by other algorithms were input into the network after normalization, and the training results are reported in **Table 4**. By comparing the results in the table, it is evident that the modeling effect obtained by combining the SCARS filtering algorithm was the best.

**Table 4:** The prediction results of the BPNN model based on THz spectroscopy.

Algorithm for band screening	RMSEC (%)	*R*_c_^2^	RMSEP (%)	*R*_p_^2^
iPLS	0.2641	0.8114	0.2851	0.8024
UVE	0.2148	0.8322	0.2363	0.8187
SCARS	0.1721	0.8447	0.1843	0.8375

**Figure 18:**
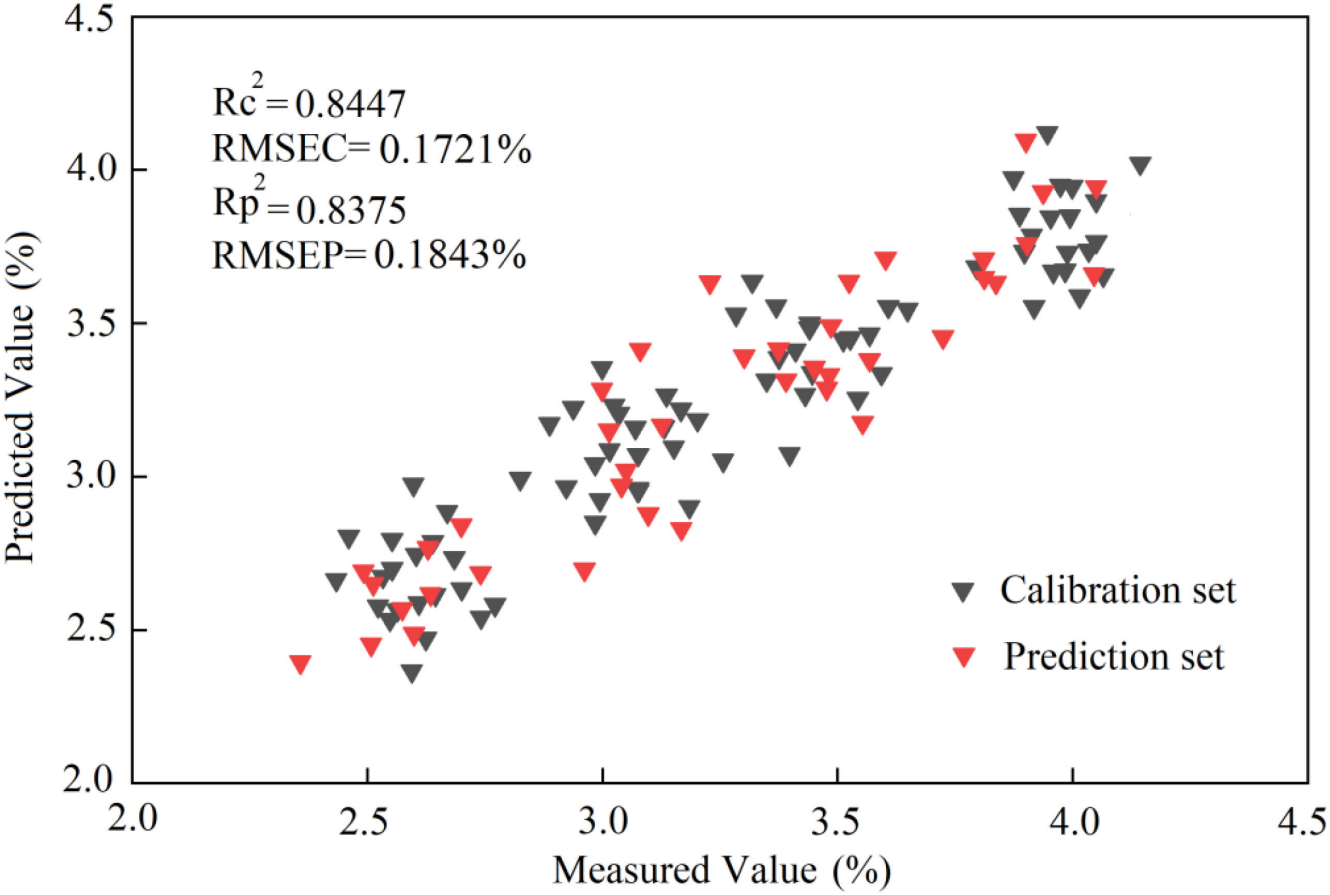
The scatter plot of the prediction results of the BPNN model based on THz spectroscopy.

According to the results reported in **Table 4**, when the BPNN algorithm was combined with the characteristic bands screened by SCARS, the RMSE values of the correction and prediction sets were respectively 0.1721% and 0.1843%, and the *R*^2^ values of the correction and prediction sets were respectively 0.8447 and 0.8375. The scatter plot of the optimal results of the BPNN model is shown in **Figure 18**.

The analysis of the nitrogen detection model based on the THz spectrum shows that the models built by the two algorithms had good prediction effects. In terms of accuracy, the RBFNN model was slightly better than the BPNN model.

## 4 Conclusion

In this study, a method for the detection of the nitrogen content of tomato based on THz spectroscopy was investigated. The basic contents of the method include data preprocessing, sample set division, characteristic frequency band screening, and model establishment. The research conclusions are as follows.

(1) In combination with the S-G smoothing algorithm, the original THz spectral data were denoised to remove invalid and interference information. The advantages and disadvantages of the KS and RS sample set partitioning algorithms were compared and analyzed. By analyzing the results of sample partitioning, it was concluded that the sample set divided by the KS algorithm could effectively cover the entire sample range, and the partitioning effect was the best.
(2) After noise reduction, the THz spectral data still relied on more low-correlation information. To improve the accuracy and efficiency of the model, the candidate characteristic bands of the THz spectrum were screened by using the UVE, SCARS, and iPLS algorithms, respectively.
(3) Based on the characteristic frequency bands screened by the iPLS, UVE, and SCARS algorithms, the RBFNN and BPNN algorithms were used to establish the models for the detection of the nitrogen content of tomatoes. The RMSEC and RMSEP values of the BPNN model were respectively 0.1722% and 0.1843%, and the determination *R_c_*^2^ and *R_p_*^2^ values were respectively 0.8447 and 0.8375. The RMSEC and RMSEP values of the RBFNN were respectively 0.1322% and 0.1855%, and its *R_c_*^2^ and *R_p_*^2^ values were respectively 0.8714 and 0.8463. Thus, the accuracy of the model established by the RBFNN algorithm was slightly higher.

## Data availability statement

The original contributions presented in the study are included in the article/supplementary material. Further inquiries can be directed to the corresponding author.

## Author contributions

Conceptualization, XZ, HG and XW; methodology, XZ, HG, LH and XW; formal analysis, CD, HG and YW data curation, XZ and CD writing—original draft preparation, XZ, YW and HG writing—review and editing, XZ and Y W. All authors contributed to the article and approved the submitted version.
